# A Novel ApoB/ApoA1 Ratio-Integrated Nomogram to Predict Cardiogenic Shock After Acute Myocardial Infarction

**DOI:** 10.31083/RCM46493

**Published:** 2026-04-21

**Authors:** Xinying Zhang, Liwen Chen, Nailiang Tian, Haidong Qin, Lei Bao

**Affiliations:** ^1^Department of Emergency, Nanjing First Hospital, Nanjing Medical University, 210006 Nanjing, Jiangsu, China; ^2^Department of Cardiology, Nanjing First Hospital, Nanjing Medical University, 210006 Nanjing, Jiangsu, China

**Keywords:** cardiogenic shock, acute myocardial infarction, ApoB/ApoA1 ratio, nomogram

## Abstract

**Background:**

Despite advances in treatment, cardiogenic shock (CS) remains a highly lethal complication of acute myocardial infarction (AMI), with mortality rates still exceeding 40%. Early identification of high-risk patients is critical, yet existing risk-stratification tools lack precision, particularly in integrating novel biomarkers such as the apolipoprotein B/A1 (ApoB/ApoA1) ratio, which reflects atherogenic lipid imbalance and has shown predictive value in cardiovascular disease.

**Methods:**

This retrospective cohort study included patients admitted with an acute coronary syndrome between December 2022 and July 2025.

**Results:**

Using the least absolute shrinkage and selection operator (LASSO) regression, a predictive nomogram was developed incorporating eight independent predictors: heart rate, respiratory rate, systolic blood pressure, white blood cell count, D-dimer, albumin, glucose, and the ApoB/ApoA1 ratio. The model demonstrated strong discriminatory performance, with an area under the curve (AUC) of 0.839 in the training cohort and 0.832 in the validation cohort. Calibration and decision curve analyses further supported the clinical utility of the nomogram.

**Conclusions:**

The inclusion of the ApoB/ApoA1 ratio, a marker associated with endothelial dysfunction, plaque instability, and metabolic dysregulation, adds significant prognostic value beyond conventional parameters. This nomogram provides a practical tool for early risk stratification, potentially guiding timely interventions and improving outcomes in high-risk patients with AMI.

## 1. Introduction

Cardiogenic shock (CS) remains the most lethal complication of an acute myocardial infarction (AMI), with mortality rates exceeding 40% despite advances in revascularization and mechanical circulatory support [1, 2]. Early identification of high-risk patients is critical for timely intervention, yet existing risk stratification tools lack precision, particularly in integrating novel biomarkers that may enhance predictive accuracy [3, 4, 5]. The apolipoprotein (Apo) B/ApoA1 ratio, a marker of atherogenic lipid imbalance, has emerged as a promising candidate for risk assessment in cardiovascular diseases [6, 7]. Recent Mendelian randomization studies have demonstrated a significant causal relationship between the ApoB/ApoA1 ratio and cardiometabolic disorders, highlighting its potential role in mediating adverse cardiovascular outcomes [6]. Furthermore, the ratio’s incremental predictive value beyond conventional lipid measures suggests its utility in refining risk models for acute cardiovascular events [6].

The pathophysiological rationale for incorporating the ApoB/ApoA1 ratio into CS prediction lies in its association with endothelial dysfunction, plaque instability, and systemic inflammation—key contributors to hemodynamic collapse in AMI [6, 7]. While traditional risk factors such as hemodynamic instability, elevated cardiac biomarkers, and multivessel disease have been extensively studied in AMI-related CS [8, 9, 10], lipid metabolism disturbances remain underexplored in this context. Notably, oxidative stress, which is exacerbated in CS, may further amplify the atherogenic effects of an elevated ApoB/ApoA1 ratio, creating a vicious cycle of myocardial injury and microvascular dysfunction [7]. Prior studies have largely focused on acute-phase reactants and hemodynamic parameters, however, the integration of lipid-derived biomarkers could provide a more comprehensive assessment of CS risk [3, 11, 12].

Clinical prediction models for CS in AMI have predominantly relied on readily available clinical and laboratory variables, but their performance remains sub-optimal, with limited external validation [3, 4, 13]. The development of a robust nomogram incorporating the ApoB/ApoA1 ratio alongside routine markers (e.g., hemodynamic indices, inflammatory markers, and coagulation parameters) may address this gap by improving discriminative ability and clinical utility [2, 5, 14]. Such a tool could facilitate early triage, guide resource allocation for advanced therapies (e.g., mechanical circulatory support), and ultimately reduce mortality [15, 16]. The CULPRIT-SHOCK trial underscored the need for personalized risk stratification to optimize revascularization strategies in AMI-CS, further justifying the exploration of biomarker-enhanced models [3, 10]. This study aims to bridge these gaps by developing and validating a novel nomogram that utilizes the ApoB/ApoA1 ratio’s unique prognostic value, thereby offering a pragmatic tool for clinicians managing high-risk AMI populations [11, 17].

## 2. Materials and Methods

### 2.1 Study Design

**Fig. 1.**
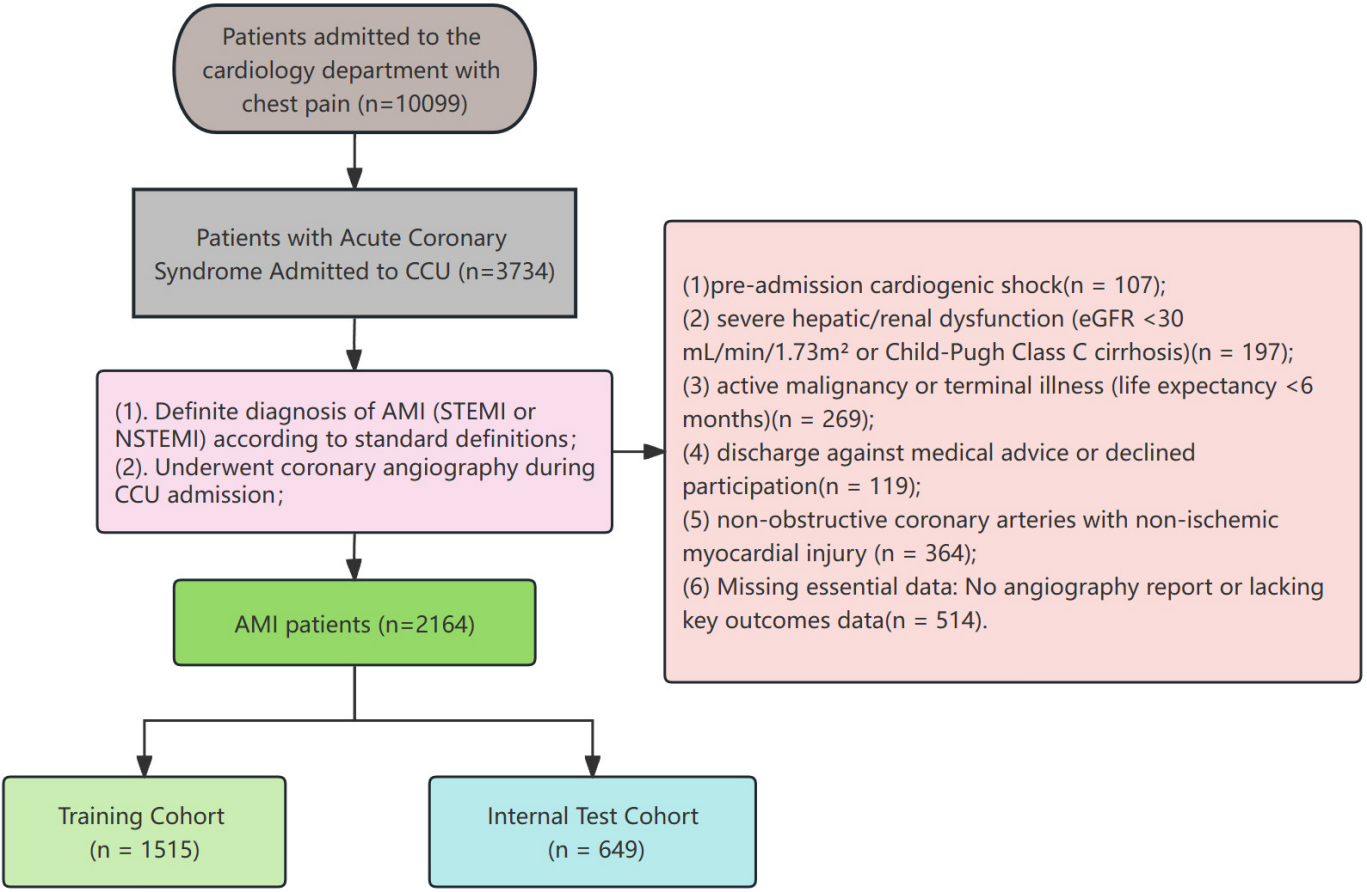
**Flowchart of patient enrollment and cohort allocation**. Abbreviations: ACS, acute coronary syndrome; AMI, acute myocardial infarction; CCU, coronary care unit; eGFR, estimated glomerular filtration rate; NSTEMI, non-ST-segment elevation myocardial infarction; STEMI, ST-segment elevation myocardial infarction.

This retrospective cohort study included consecutive patients admitted to the Coronary Care Unit (CCU) of the Nanjing First Hospital for chest pain between December 2022 and July 2025. Inclusion criteria included: (1) age ≥18 years; (2) meeting diagnostic criteria for acute coronary syndrome (ACS) including ST-segment elevation myocardial infarction (STEMI), non-ST-segment elevation myocardial infarction (NSTEMI), or unstable angina [18]; and (3) undergoing invasive coronary angiography during the index hospitalization to confirm the coronary pathology. Exclusion criteria included: (1) pre-admission cardiogenic shock; (2) severe hepatic/renal dysfunction (estimated glomerular filtration rate (eGFR) <30 mL/min/1.73 m^2^ or Child-Pugh Class C cirrhosis); (3) active malignancy or terminal illness (life expectancy <6 months); (4) discharge against medical advice or declined participation; (5) non-obstructive coronary arteries with non-ischemic myocardial injury; (6) Missing essential data: No angiography report or lacking key outcomes data. The detailed patient selection process is shown in Fig. 1. The primary endpoint was post-AMI CS, which was defined as sustained hypotension (systolic blood pressure (SBP) <90 mmHg) accompanied by clinical signs of hypoperfusion and adequate cardiac filling status, for which an intra-aortic balloon pump (IABP) implantation was recommended [19].

### 2.2 Data Collection

Data were extracted from electronic medical record systems, including baseline demographic and clinical characteristics, laboratory results from fasting venous blood samples collected immediately upon admission, and clinical follow-up events. Demographic data included gender, age, and body mass index (BMI). Clinical characteristics included a history of cardiovascular and cerebrovascular diseases, and laboratory results.

### 2.3 Statistical Analysis

The dataset collected from the Nanjing First Hospital was randomly divided into training and validation sets in a 7:3 ratio, and baseline characteristics were compared between cohorts. Continuous variables following normal distribution were expressed as mean ± standard deviation (SD), while non-normally distributed data were presented as median with an interquartile range (IQR). In univariable analyses, categorical variables were assessed using the Chi-square test or Fisher’s exact test, and continuous variables were analyzed using Student’s *t*-test or Mann-Whitney U test as appropriate. Within the training cohort, least absolute shrinkage and selection operator (LASSO) regression was employed to screen for predictors of cardiogenic shock, followed by the construction of a predictive nomogram. A two-tailed *p*-value < 0.05 was considered statistically significant. All analyses were performed using R software (version 4.2.2; R Foundation for Statistical Computing, Vienna, Austria) and MSTATA (www.mstata.com).

### 2.4 Model Evaluation

Model performance was evaluated by receiver operating characteristic (ROC) curve analysis, with the area under the curve (AUC) ranging from 0.5 (no discriminative ability) to 1.0 (perfect discrimination). Calibration curves were generated to assess agreement between predicted and observed outcomes. Decision curve analysis (DCA) was further conducted to determine the clinical net benefit threshold of the prediction model.

## 3. Results

### 3.1 Patient Characteristics

As shown in Table 1, this study analyzed baseline characteristics in a training cohort (N = 1515) and an internal test cohort (N = 649). No significant differences existed in gender distribution (male: 79.8% vs 81.7%, *p* = 0.318), age (64 ± 13 vs 63 ± 12 years, *p* = 0.810), or BMI (24.8 (22.6, 27.0) vs 24.6 (22.6, 26.8) kg/m^2^, *p* = 0.454). Vital signs including heart rate, respiratory rate, and blood pressure, showed comparable values (all *p* > 0.40). Disease characteristics revealed a similar prevalence of STEMI (51.2% vs 51.8%, *p* = 0.814) and comorbidities: hypertension (45.6% vs 47.5%, *p* = 0.430), diabetes (32.1% vs 34.1%, *p* = 0.386), stroke history (9.6% vs 8.2%, *p* = 0.299), and atrial fibrillation (2.6% vs 3.2%, *p* = 0.390). Laboratory analysis identified a significantly higher neutrophil count in the test cohort (7.7 ± 3.3 vs 8.0 ± 3.9 × 10^9^/L, *p* = 0.046). Other parameters including white blood cells, hemoglobin, liver/kidney function, and comprehensive lipid profiles showed no significant differences (all *p* > 0.05). The cohorts demonstrated substantial baseline consistency for predictive modeling.

**Table 1.** **Patient demographics and baseline characteristics**.

Characteristic		Cohort		*p*-value
		Training cohort	Internal test cohort	
		N = 1515	N = 649	
Gender, n (%)				0.318
	Female	306 (20.2%)	119 (18.3%)	
	Male	1209 (79.8%)	530 (81.7%)	
Age, years		64 ± 13	63 ± 12	0.810
BMI, kg/m^2^		24.8 (22.6, 27.0)	24.6 (22.6, 26.8)	0.454
HR, bpm		81 ± 16	82 ± 17	0.655
RR, breaths/min		17.5 ± 3.3	17.5 ± 3.4	0.925
SBP, mmHg		137 ± 23	136 ± 23	0.406
DBP, mmHg		84 ± 15	84 ± 15	0.955
Smoking, n (%)		777 (51.3%)	347 (53.5%)	0.352
STEMI, n (%)		776 (51.2%)	336 (51.8%)	0.814
Hypertension, n (%)		691 (45.6%)	308 (47.5%)	0.430
Stroke, n (%)		145 (9.6%)	53 (8.2%)	0.299
Type 2 diabetes, n (%)		487 (32.1%)	221 (34.1%)	0.386
Atrial fibrillation, n (%)		39 (2.6%)	21 (3.2%)	0.390
WBC, ×10^9^/L		9.7 ± 3.4	10.1 ± 4.1	0.060
Neutrophil count, ×10^9^/L		7.7 ± 3.3	8.0 ± 3.9	0.046
Lymphocyte count, ×10^9^/L		1.40 ± 0.66	1.40 ± 0.61	0.767
Hemoglobin, g/L		135 ± 20	136 ± 20	0.250
Platelet count, ×10^9^/L		209 ± 66	209 ± 67	0.928
D-dimer, mg/L		0.42 (0.23, 0.73)	0.42 (0.23, 0.80)	0.557
ALT, U/L		31 (20, 51)	31 (20, 49)	0.734
AST, U/L		63 (29, 157)	63 (28, 162)	0.909
Albumin, g/L		38.6 ± 3.8	38.6 ± 3.8	0.672
Urea, mmol/L		7.0 ± 4.0	7.1 ± 3.9	0.601
Creatinine, µmol/L		77 (66, 93)	77 (66, 96)	0.493
Uric acid, µmol/L		369 ± 118	368 ± 112	0.964
Glucose, mmol/L		7.25 ± 2.80	7.48 ± 3.16	0.120
Triglycerides, mmol/L		1.89 ± 1.39	1.97 ± 1.52	0.231
Total cholesterol, mmol/L		4.52 ± 1.21	4.52 ± 1.20	0.918
HDL-C, mmol/L		0.94 ± 0.21	0.93 ± 0.22	0.215
LDL-C, mmol/L		2.62 ± 0.90	2.61 ± 0.92	0.867
Apolipoprotein A1 (ApoA1), g/L		1.20 ± 0.21	1.19 ± 0.22	0.821
Apolipoprotein B (ApoB), g/L		0.89 ± 0.29	0.90 ± 0.30	0.569
ApoB/ApoA1 ratio		1.50 ± 0.64	1.50 ± 0.69	0.921
Lipoprotein(a), mg/L		33 (15, 84)	36 (15, 85)	0.402

Abbreviations: BMI, body mass index; HR, heart rate; RR, respiratory rate; SBP/DBP, systolic/diastolic blood pressure; STEMI, ST-elevation myocardial infarction; WBC, white blood cell count; ALT, alanine aminotransferase; AST, aspartate aminotransferase; HDL-C/LDL-C, high-/low-density lipoprotein cholesterol; ApoB/ApoA1, apolipoprotein B/apolipoprotein A1.

### 3.2 Predictive Model

**Fig. 2.**
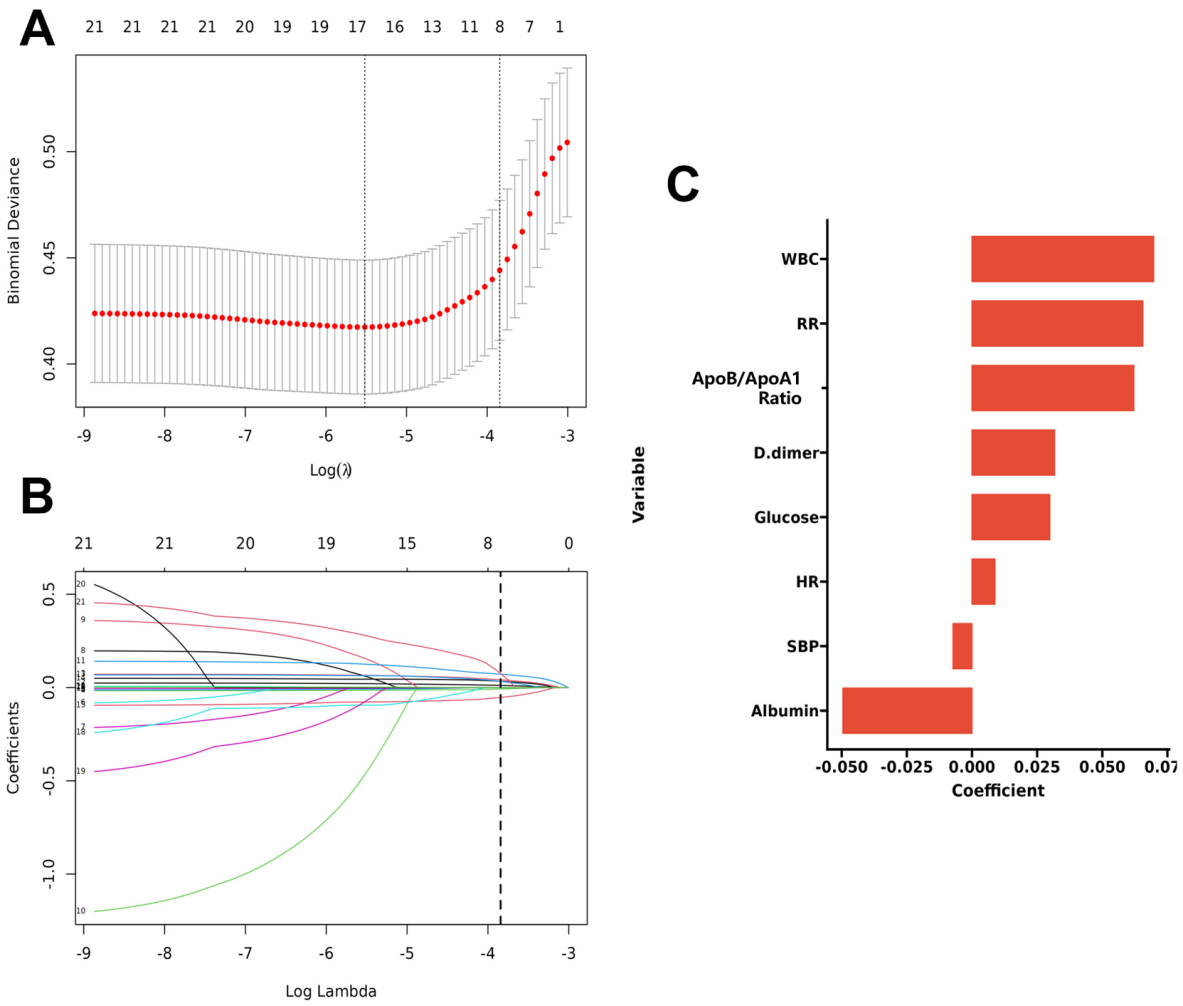
**LASSO regression analysis for variable selection**. (A) Binomial Deviance vs. Log(λ) in LASSO Cross-Validation. (B) LASSO Coefficient Trajectories Across Log(λ). (C) Forest Plot of Selected LASSO Coefficients. Abbreviations: LASSO, least absolute shrinkage and selection operator; λ, regularization parameter; WBC, white blood cell count; RR, respiratory rate; D-dimer, D-dimer level; SBP, systolic blood pressure; ApoB/ApoA1, apolipoprotein B/apolipoprotein A1; HR, heart rate.

Candidate predictor variables included body mass index (BMI), heart rate (HR), respiratory rate (RR), SBP, STEMI, hypertension, type 2 diabetes, stroke, atrial fibrillation, white blood cell count (WBC), hemoglobin level, platelet count, D-dimer, albumin, creatinine, glucose, low-density lipoprotein cholesterol, apolipoprotein A1, apolipoprotein B, ApoB/ApoA1 ratio, and lipoprotein(a). Following their inclusion in the initial model, LASSO regression analysis applied to the training cohort ultimately identified eight potential predictors. **Supplementary Table 1** displays the regression coefficients for each variable. As shown in Fig. 2, Fig. 2A,B illustrate LASSO cross-validation to select the optimal λ, balancing model fit and parsimony. Fig. 2C highlights the magnitude and direction of coefficients for variables retained in the final model.

**Fig. 3.**
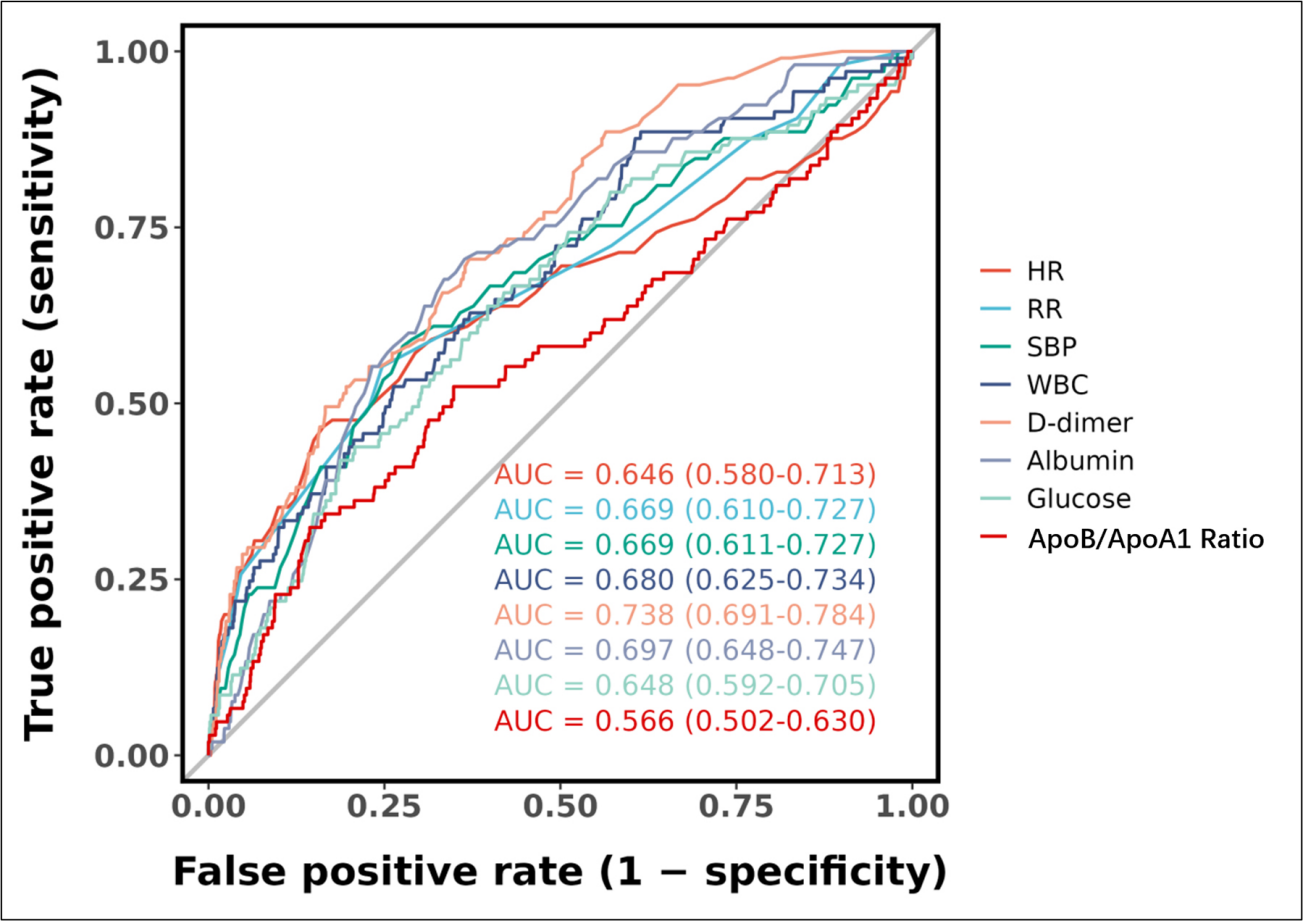
**Receiver operating characteristic (ROC) curves for individual predictive variables**. Abbreviations: AUC, area under the curve; CI, confidence interval; HR, heart rate; RR, respiratory rate; SBP, systolic blood pressure; WBC, white blood cell count; ApoB/ApoA1 ratio, apolipoprotein B/apolipoprotein A1 ratio.

**Fig. 4.**
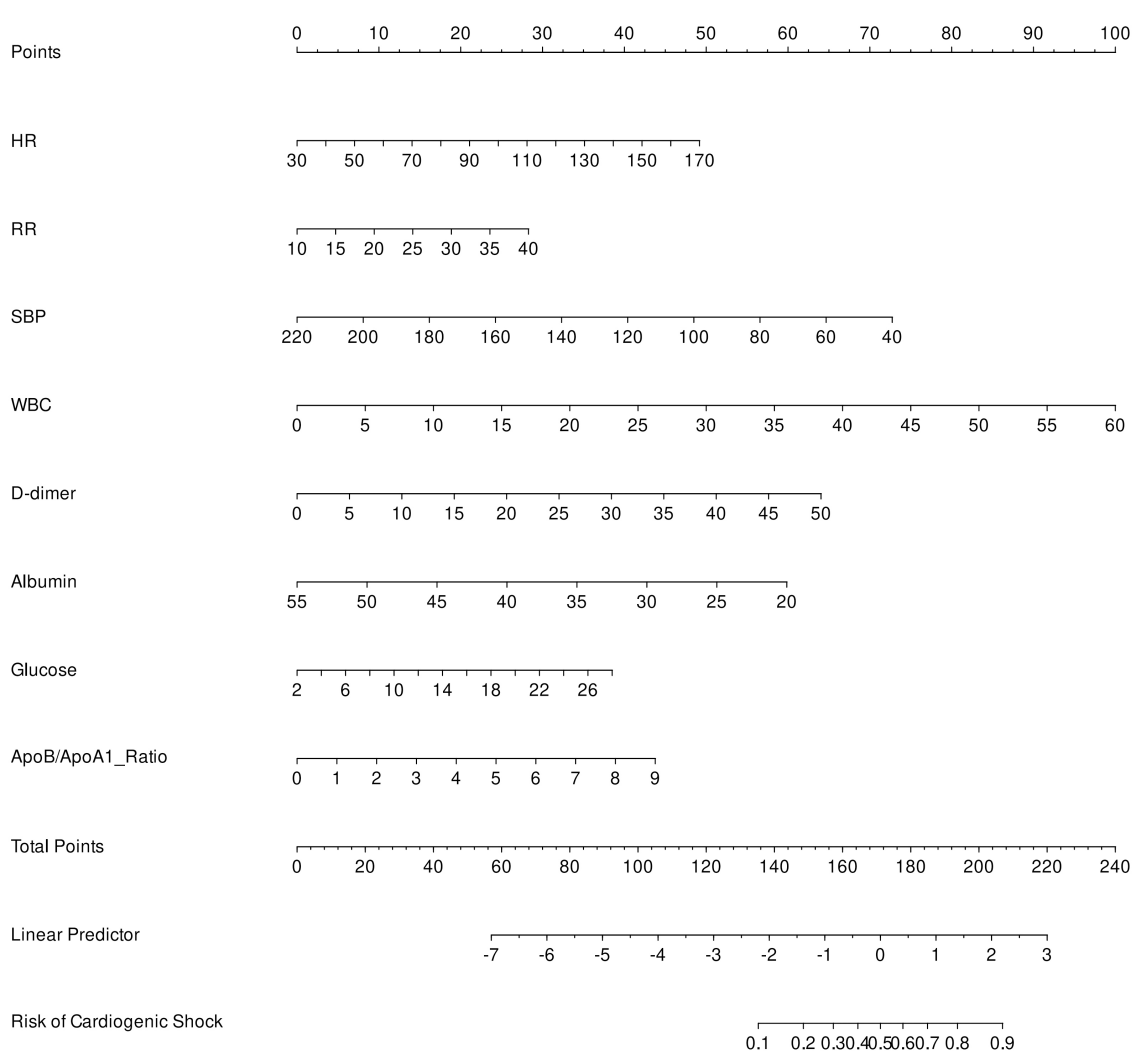
**Nomogram for predicting the risk of post-myocardial infarction cardiogenic shock**. Abbreviations: HR, Heart Rate; RR, respiratory rate; SBP, systolic blood pressure; WBC, white blood cell count; ApoB/ApoA1 ratio, apolipoprotein B/apolipoprotein A1 ratio.

As shown in Fig. 3, the ROC analysis of the above-mentioned variables yielded AUC values greater than 0.5. The ROC curve analysis demonstrated the discriminative performance of individual variables in predicting the outcome event (cardiogenic shock), with D-dimer exhibiting the highest predictive value (AUC = 0.738, 95% CI: 0.691–0.784), followed by albumin (AUC = 0.697, 95% CI: 0.648–0.747) and WBC (AUC = 0.680, 95% CI: 0.625–0.734). SBP and RR showed comparable predictive accuracy (AUC = 0.669, 95% CI: 0.611–0.727 and AUC = 0.669, 95% CI: 0.610–0.727, respectively), while HR and glucose displayed moderate discrimination (AUC = 0.646, 95% CI: 0.580–0.713 and AUC = 0.648, 95% CI: 0.592–0.705, respectively). The ApoB/ApoA1 ratio demonstrated the lowest predictive capacity among all examined variables (AUC = 0.566, 95% CI: 0.502–0.630). These results indicate varying degrees of predictive utility across different physiological and biochemical parameters for the outcome event. The final logistic model included 8 independent predictors (HR, RR, SBP, WBC, D-dimer, albumin, glucose, and ApoB/ApoA1 ratio) and was developed as a simple-to-use nomogram, which is illustrated in Fig. 4. An additional multivariate logistic regression analysis was performed in the training cohort, with the results presented in Table 2. As demonstrated in Table 2, multivariable logistic regression identified significant predictors of cardiogenic shock: positive associations with HR (OR 1.02, 95% CI: 1.01–1.03), RR (OR 1.10, 95% CI: 1.04–1.16), WBC (OR 1.12, 95% CI: 1.06–1.18), D-dimer (OR 1.06, 95% CI: 1.02–1.11), glucose (OR 1.08, 95% CI: 1.02–1.15), and ApoB/ApoA1 ratio (OR 1.53, 95% CI: 1.16–2.02) (all *p* < 0.05), and negative associations with SBP (OR 0.98, 95% CI: 0.97–0.99) and albumin (OR 0.90, 95% CI: 0.85–0.95) (both *p* < 0.001). The final logistic model, incorporating 8 independent predictors, was transformed into a user-friendly nomogram, as shown in Fig. 4.

**Table 2.** **Results of multivariate logistic regression for training cohort**.

Characteristic	N	Event N	OR	95% CI	*p*-value
HR	1515	105	1.02	1.01, 1.03	0.002
RR	1515	105	1.10	1.04, 1.16	<0.001
SBP	1515	105	0.98	0.97, 0.99	<0.001
WBC	1515	105	1.12	1.06, 1.18	<0.001
D-dimer	1515	105	1.06	1.02, 1.11	0.010
Albumin	1515	105	0.90	0.85, 0.95	<0.001
Glucose	1515	105	1.08	1.02, 1.15	0.014
ApoB/ApoA1 Ratio	1515	105	1.53	1.16, 2.02	0.003

Abbreviations: OR, odds ratio; CI, confidence interval; HR, heart rate; RR, respiratory rate; SBP, systolic blood pressure; WBC, white blood cell count; ApoB/ApoA1, apolipoprotein B/apolipoprotein A1.

### 3.3 Model Performance

**Fig. 5.**
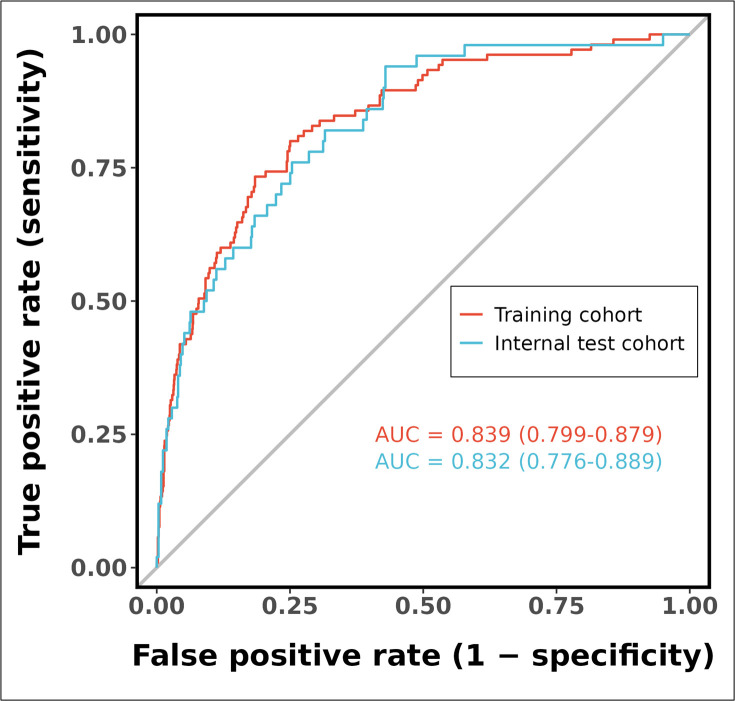
**Receiver operating characteristic (ROC) curves of the predictive model in training and internal test cohorts**. Abbreviations: AUC, area under the curve; CI, confidence interval.

**Fig. 6.**
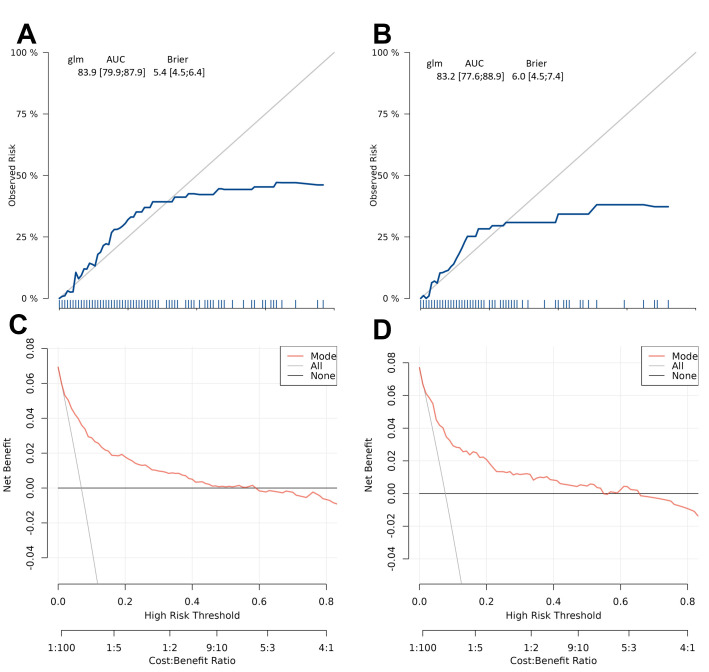
**Calibration and clinical utility of the risk prediction model in training and validation cohorts**. The model demonstrates reasonable calibration (blue line generally follows the diagonal with minor deviations in low-probability ranges) in both training (A) and test (B) cohorts, suggesting predicted risks show acceptable correspondence with observed outcomes. Decision curve analysis confirms the model provides meaningful net benefit across a range of high-risk thresholds, supporting its clinical utility for risk stratification in both training (C) and test (D) cohorts. Abbreviations: AUC, area under the curve; CI, confidence interval.

As shown in Fig. 5, the ROC curve analysis demonstrated that the predictive model achieved an AUC of 0.839 (95% CI: 0.799–0.879) for discriminating cardiogenic shock in the training cohort, indicating robust discriminative performance. Similarly, in the validation cohort, the model maintained excellent predictive accuracy with an AUC of 0.832 (95% CI: 0.776–0.889), showing consistent generalization across different datasets. The overlapping confidence intervals between the training and validation cohorts suggest stable model performance without significant degradation in predictive capability when applied to independent datasets. The calibration curves in Fig. 6A,B indicate moderate agreement between observed and predicted cardiogenic shock probabilities across cohorts, suggesting the nomogram may have reasonable predictive validity within this study population. The curves closely align with the ideal line, indicating robust predictive accuracy. As shown in Fig. 6C,D, DCA reveals significant net clinical benefit within the applicable threshold probability range, supporting the model’s clinical utility despite potential physician interpretation errors at high-risk thresholds.

## 4. Discussion

This study presents a novel prognostic nomogram incorporating the ApoB/ApoA1 ratio alongside established clinical and biochemical markers to predict the risk of cardiogenic shock in patients with acute myocardial infarction. The model demonstrated high discriminatory accuracy, precise calibration, and provided significant clinical net benefit based on DCA.

The multimodal nomogram developed in this study significantly improves upon traditional CS risk models by integrating inflammatory, nutritional, and lipid metabolic markers with conventional hemodynamic parameters. Traditional CS risk stratification systems such as the IABP-SHOCK II score (age >73 years, prior stroke, glucose >10.6 mmol/L, creatinine >1.5 mg/dL, lactate >5 mmol/L, TIMI flow <3) focused predominantly on hemodynamic and metabolic parameters [3, 20, 21, 22]. In contrast, this model uniquely integrates inflammatory (WBC, D-dimer), nutritional (albumin), and novel lipid metabolism markers (ApoB/ApoA1 ratio), providing a more comprehensive pathophysiological assessment. The SCAI shock classification system, while valuable for predicting mortality, lacks the incorporation of specific biomarkers and primarily relies on clinical signs of hypoperfusion [20, 23]. The CardShock risk score (age, prior AMI/CABG, confusion, ACS etiology, LVEF <40%, lactate >2 mmol/L) similarly lacks inflammatory and lipid markers [24]. Recent machine learning approaches have incorporated broader variables but often lack clinical interpretability [5]. This model’s inclusion of respiratory rate and D-dimer aligns with emerging recognition of pulmonary dysfunction and coagulopathy in CS pathophysiology [9, 25], while albumin levels reflect pathophysiological disturbances and predict adverse outcomes in CS [26]. In our derivation cohort, the nomogram showed numerically higher discrimination than the adapted CULPRIT-SHOCK score [3] (internally validated AUC 0.84 vs. 0.72), though this observation requires cautious interpretation due to fundamental cohort differences. The integration of multimodal biomarkers may contribute to performance variations across prediction tools.

The incorporation of the ApoB/ApoA1 ratio into predictive models for CS following AMI has substantial clinical significance, as evidenced by extensive research on its pathophysiological and prognostic implications. Mendelian randomization studies have established a causal relationship between elevated ApoB/ApoA1 ratios and cardiometabolic diseases (CMD), including ischemic heart disease and major adverse cardiovascular events (*p* < 0.05), with the ratio mediating hemoglobin A1c, fasting insulin levels, and other metabolic risk factors [6]. The ratio’s superior predictive value over conventional lipid parameters is demonstrated by its strong association with macrovascular complications (HR 1.19, 95% CI: 1.06–1.34) and the risk of myocardial infarction [27], while traditional markers like LDL cholesterol showed weaker correlations. The ApoB/ApoA1 ratio emerges as the best lipid predictor for ischemic stroke [28] and cardiovascular mortality (OR 2.13, 95% CI: 1.48–3.07) [29], with longitudinal data revealing its predictive capacity decades before the onset of events [30]. These findings are particularly relevant given that lipid metabolism disturbances in AMI-CS involve both quantitative (reduced HDL/ApoA1) and qualitative (pro-inflammatory LDL modifications) abnormalities [31]. The current study’s demonstration of the ratio’s independent predictive value (OR 1.53) aligns with multi-omic analyses identifying high ApoB/ApoA1 ratios as biomarkers of increased cardiometabolic risk [32]. Importantly, the ratio’s predictive superiority over isolated lipid parameters [33] and its modification through dietary interventions [34] underscore its clinical utility for both risk stratification and therapeutic monitoring in AMI-CS patients. Despite the prognostic value of ApoB/ApoA1, its clinical utility in emergency AMI settings is currently constrained by assay turnaround times (2–4 hours). Development of point-of-care platforms is warranted for acute care implementation.

This study provides three significant advancements: it addresses critical gaps in existing CS risk models by incorporating underutilized yet pathophysiologically relevant markers [3, 6, 20]; establishes the ApoB/ApoA1 ratio as a novel predictor capturing residual lipid risk beyond conventional parameters [6, 27]; and demonstrates superior discriminative performance relative to prior scores [3, 23, 35], while maintaining clinical practicality. By integrating readily obtainable ICU parameters with advanced biomarkers, our model creates a translational bridge between emergent risk stratification [23, 36] and approaches to personalized medicine [5, 37]. Prospective validation should assess performance across SCAI stages [20, 37] and CS phenotypes [37], particularly given the ratio’s potential mediation of metabolic dysregulation [6]. Future research must explore whether ratio-targeted interventions (e.g., ApoA1 infusion, PCSK9 inhibitors) could modify CS risk [34], potentially opening new therapeutic frontiers for this lethal condition.

## 5. Limitations

This study has several limitations that warrant consideration. As a single-center observational study, the generalizability of findings may be influenced by institution-specific clinical practices and potential selection bias inherent in non-randomized designs. While internal validation demonstrated robust model performance, the generalizability of our nomogram requires further evaluation through external validation across diverse healthcare settings with varying resource availability, particularly regarding standardized ApoB/ApoA1 ratio measurement protocols. These considerations highlight important directions for future multicenter validation studies.

## 6. Conclusions

This study establishes a practical prediction tool for post-MI cardiogenic shock requiring IABP support by combining routine clinical signs (HR, RR, SBP) and key biomarkers (D-dimer, glucose, ApoB/ApoA1 ratio, albumin, WBC) into a nomogram. It enables early risk stratification, facilitating timely interventions to mitigate the severity of shock and to improve survival in this critical population.

## Availability of Data and Materials

The data from this study are available from the corresponding author upon reasonable request.

## Supplementary Material

Supplementary material associated with this article can be found, in the online version, at https://doi.org/10.31083/RCM46493.
