# Supplementary material for: A Novel ApoB/ApoA1 Ratio-Integrated Nomogram to Predict Cardiogenic Shock After Acute Myocardial Infarction
Source: Rev Cardiovasc Med. 2026 Apr 21;27(4):46493. doi: 10.31083/RCM46493 (PMC13155974; doi:10.31083/RCM46493)
Supplement: Supplementary file 1 [file 2153-8174-27-4-46493-s1.zip › Supplymentary Table 1.docx]

**Supplymentary Table 1:** The coefficients of Lasso regression analysis

| **variable** | Coefficient |
| --- | --- |
| **(Intercept)** | -2.716907547 |
| **BMI** | 0.000000000 |
| **HR** | 0.008810611 |
| **RR** | 0.065581648 |
| **SBP** | -0.007279031 |
| **STEMI** | 0.000000000 |
| **Hypertension** | 0.000000000 |
| **Type 2 diabetes** | 0.000000000 |
| **Stroke** | 0.000000000 |
| **Atrial Fibrillation** | 0.000000000 |
| **WBC** | 0.069715072 |
| **Hemoglobin level** | 0.000000000 |
| **Platelet count** | 0.000000000 |
| **D-dimer** | 0.031714635 |
| **Albumin** | -0.049550360 |
| **Creatinine** | 0.000000000 |
| **Glucose** | 0.029861902 |
| **LDL-C** | 0.000000000 |
| **Apolipoprotein A1** | 0.000000000 |
| **Apolipoprotein B** | 0.000000000 |
| **ApoB/ApoA1 Ratio** | 0.062131364 |
| **Lipoprotein(a)** | 0.000000000 |

BMI:Body Mass Index; HR:Heart Rate; RR:Respiratory Rate; SBP:Systolic Blood Pressure; STEMI:ST-Elevation MyocardialInfarction; WBC:White Blood Cell Count; LDL-C:Low-Density Lipoprotein Cholesterol; ApoB/ApoA1:Apolipoprotein B/Apolipoprotein A1
